# Impact of a “vegetables first” approach to complementary feeding on later intake and liking of vegetables in infants: a study protocol for a randomised controlled trial

**DOI:** 10.1186/s13063-021-05374-7

**Published:** 2021-07-26

**Authors:** Jeanette P. Rapson, Pamela R. von Hurst, Marion M. Hetherington, Cathryn A. Conlon

**Affiliations:** 1 School of Sport, Exercise and Nutrition, Massey University, Auckland, New Zealand; 2 School of Psychology, University of Leeds, Leeds, England, UK

**Keywords:** Infants, Weaning, Introducing vegetables, Food preference, Vegetable intake

## Abstract

**Background:**

Vegetables as first complementary foods for infants may programme taste preferences that lead to improved vegetable intake in children. Yet few studies have investigated the impact of a ”vegetables first” approach to complementary feeding, especially in New Zealand. The purpose of this randomised control trial is to investigate the effect of starting complementary feeding with vegetables only on infants’ later intake and liking of vegetables, compared to those starting with fruit and vegetables.

**Methods/design:**

One-hundred and twenty mother-infant pairs living in Auckland, New Zealand, will be randomised to receive either vegetables only (intervention) or fruit and vegetables (control) for 28 days, starting from the first day of complementary feeding at around 4–6 months of age. Infants will be presented with a brassica (broccoli), followed by a green leafy vegetable (spinach) and sweet fruit (pear) at 9 months of age. The primary outcome measures of intake of each food will be assessed using a weighed food diary. Secondary outcome measures of overall intake, liking and wanting of vegetables will be assessed using a food frequency questionnaire, liking tool and video coding tool, respectively, at 9, 12, and 24 months of age. Infant growth and iron status will be assessed as part of health screening and monitoring at baseline, post intervention and 9 months of age. Other biological samples to be collected include infant stool samples, vitamin D (mother and infant), iron status (mother), and mothers’ diet.

**Discussion:**

This randomised, controlled trial will be the first to our knowledge to investigate a “vegetables first” approach to complementary feeding on infants’ liking and intake of vegetables in New Zealand. Comparison against standard practice (fruit and vegetables as first foods) should complement other trials underway, such as the Baby’s First Bites and Nordic OTIS trial. Results may contribute to the evidence supporting complementary feeding guidelines in New Zealand and worldwide.

**Trial registration:**

Australian New Zealand Clinical Trial Registry ACTRN12619000737134. Registered on 16 May 2019.

**Supplementary Information:**

The online version contains supplementary material available at 10.1186/s13063-021-05374-7.

## Background

Children are encouraged to eat plenty of different vegetables daily in order to meet a range of nutrient requirements for growth and development, immune function, and digestive health [1, 2]. Children with adequate vegetable intake may have a lower risk of obesity [3] and cardiovascular disease in adulthood [4, 5], and be more likely to develop healthy eating habits for life [6, 7]. Yet children’s vegetable consumption remains low worldwide [8–12]. In the UK, only 14% of children (5–7 years) eat the recommended five or more portions of vegetables and fruit per day [12]. Vegetable intake in the US is similarly low, with the FITS study finding 30% of children 12 months or older not consuming any vegetable servings on a given day [13]. New Zealand children (2–4 years) are doing better but intakes are still inadequate, with 46.1% and 71.2% of children consuming enough vegetables and fruit, respectively [10].

Reasons for poor vegetable consumption may relate to many factors, including a dislike of bitter taste and poor access [13–15]. Infants have an innate preference for sweetness since breastmilk is sweet and may find bitter taste, such as in dark leafy greens, particularly difficult to accept [16–18]. Meanwhile, children from food insecure households may have an additional challenge of accessing a variety of vegetables [19]. Eurostat data found 5.5% of UK households with children were unable to afford meat, fish, or a vegetarian alternative every second day [20]. In the US, an estimated 14% of US families with children experienced food insecurity in 2018 [21]. Similarly, although most New Zealand children live in food-secure households, a substantial number do not (19%) [22] and so are less likely to have vegetables purchased and offered to them [23].

It is known that offering infants a variety of vegetables from the start of complementary feeding may promote liking through familiarisation and facilitate intake throughout childhood [24–26]. However, many infants receive fruit and sweet tasting vegetables (e.g. pumpkin, sweet potato) as their first foods [27, 28]. Furthermore, most commercial infant foods are largely fruit or sweet/starchy vegetable based [29–31]. In a sample of traditionally spoon fed New Zealand infants (n = 628), 1% received “only fruits, no vegetables”, 17% “mainly fruits, some vegetables”, 46% “half fruit, half vegetables”, and 4% “only vegetables, no fruits” when solids were first introduced; just over half were consuming iron-rich foods (meat or infant cereal) at 6 months of age [32]. A recent systematic review of practices to promote vegetable acceptance in the first three years of life concluded that introducing vegetables at the beginning of complementary feeding, giving a different type of vegetable every day and ensuring repeated exposure to the same vegetable following an interval of a few days are the most promising strategies to promote vegetable intake in early childhood [25].

The prioritisation of vegetables only from the start of complementary feeding is known as a “vegetables first” approach to complementary feeding. Only a handful of studies investigating the effects of this approach have been published, with none in New Zealand [33–37]. Barends et al. compared the effects of repeated exposure and starting complementary feeding with vegetables only across four different treatment groups over 18 days. They found that starting complementary feeding with vegetables, but not with fruits, may promote vegetable acceptance and that liking correlated positively with intake. Their follow-up study [35] suggested that starting complementary feeding with vegetables only results in higher daily vegetable consumption until at least 12 months of age; however, further investigation was needed on how to maintain this effect until 24 months of age. Three other studies suggest vegetables as first foods may improve vegetable acceptance later, at least in the short term [33, 36–39]. However, comparability of findings remains limited due to considerable variability in study designs, small sample sizes, and/or poor ecological validity (e.g. conducting trials in the laboratory rather than the home environment). Standardisation across studies could be improved with the use of recent development and validation of tools to assess vegetable liking, such as the Feeding Infants: Behaviour and Facial Expression Coding System (FIBFECS) [40] or an in-home adapted elaborate method for liking tool [41].

Historically, most guidelines on complementary feeding have included vegetables as first complementary foods but alongside fruit and iron-rich foods without a prescribed sequence [1]. The systematic evidence compiled by the UK Public Health England and Scientific Advisory Committee on Nutrition (SACN) [42] however has informed the current NHS recommendations to give a single vegetable and fruit as first foods [43]. While there may be enough evidence to start recommending vegetables as first complementary foods, there is still a need for more longitudinal randomised controlled trials (RCTs) [24, 38, 44, 45]; especially those comparing infants receiving vegetables only to standard practice. Two other trials examining the effects of providing vegetables as first foods on vegetable acceptance are in progress, including the Baby’s First Bites (BFB) trial in the Netherlands and the OTIS Nordic trial. In the BFB, infants are randomised to receiving, with or without a positive feeding programme, either fruits and a sweet vegetable or a variety of vegetables. However, all infants start complementary feeding with rice-flour porridge and then vegetables as their first tastes [45]. The Nordic OTIS trial is comparing effects of the recommended, Swedish complementary diet (i.e. starting with baby cereals, commercially available baby foods) to one based on Nordic foods (i.e. increased plant foods including fruit and vegetables and reduced sweets, dairy and meat) on food acceptance [44]. Infants in the Nordic group are systematically introduced to fruits (e.g. apples, raspberry, buckthorn/lingonberry, cranberry) and vegetables (green peas, cauliflower, turnip, daikon) from the start of the intervention, rather than vegetables only.

RCTs are one of the most rigorous ways to establish cause-effect relationships between treatment and outcomes [46], and RCT data is a gold standard for translating evidence into practice [47]. Our RCT will test the hypothesis that exposure to vegetables only during the first 4 weeks of complementary feeding increases later intake and liking of vegetables in infants, compared to a control group of fruit and vegetables. If this is supported, our results could contribute to the evidence for recommendations for a “vegetables first” approach to complementary feeding in New Zealand and beyond.

### Hypothesis

Exposure to vegetables only during the first 4 weeks of complementary feeding increases later intake and liking of vegetables in infants, compared to a control group which includes both fruit and vegetables.

### Aim

The overall aim of this study is to determine whether exposure to vegetables only during the first 4 weeks of complementary feeding increases later intake and liking of vegetables in infants, compared to a control group which includes both fruit and vegetables.

## Methods/design

**Fig. 1:**
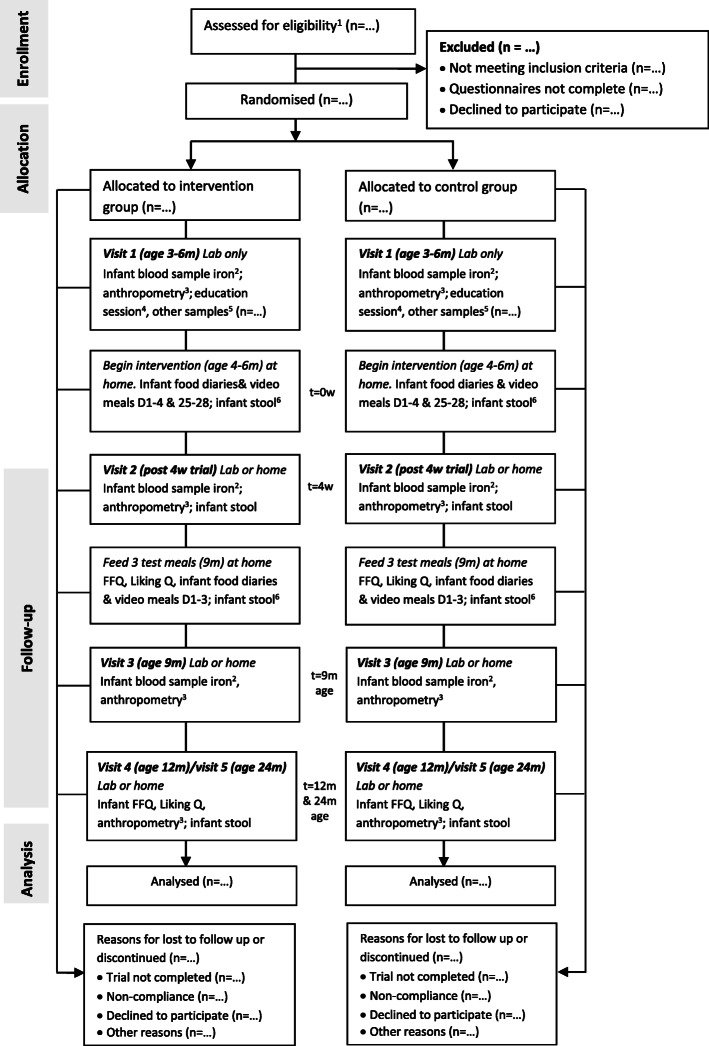
Schematic diagram of study design. ^1^Questionnaires: eligibility, demographics, baby eating behaviours, and pregnancy/lactation dietary questionnaires. ^2^Blood biomarkers: serum ferritin, C-reactive protein (CRP), and haemoglobin. ^3^Weight, head circumference, and length. ^4^Includes delivering all study materials, protocol instructions, and information on complementary feeding. ^5^Blood samples to assess the status of mother’s iron/vitamin D and infant’s vitamin D. ^6^Infant stool sample collected by mother 1 week or less before infant receives the first intervention food/test meal. Lab, laboratory (Massey University Human Nutrition Research Unit); D, day; w, week; m, month; t, time-point; FFQ, food frequency questionnaire; Q, questionnaire

This study is designed as a randomised controlled trial comparing two parallel groups following either a “vegetables first” approach to complementary feeding or standard practice of feeding fruit and vegetables. This involves a 4-week intervention period starting from the beginning of complementary feeding (between 4 and 6 months old), with the primary endpoint of vegetable intake at 9 months of age (Fig. 1). The duration of the intervention targets the sensitive period for taste development [48], is logistically feasible, and allows for a timely progression to other foods and textures (e.g. mashed) that is expected by around 7 months. An endpoint of 9 months old aims to capture effects over time without significant loss to follow-up, and enables the study foods to be retested in an age-appropriate form before infants have transitioned to family foods at 12 months of age.

### Participants

Mother-infant pairs (4 to 6 months old) living in Auckland will be invited to register on the study website (www.vegesfirststudy.co.nz) [49] advertised on social media, email, and community notice boards. Based on the findings of Barends and colleagues [35], we calculated that 65 participants (a minimum of 52 participants, and allowing for a 20% potential dropout rate) would be required for each arm of the trial to demonstrate a clinically significant difference at 80% power and 5% statistical significance. Power calculations used a 21-unit difference of vegetable intake (grammes) between intervention group and control, and a mean standard deviation of 43 and 29 units, respectively. The sample size was calculated using the formula below [50]:

$$ \mathrm{N}={2\upalpha}^2\mathrm{K}/{\left({\upmu}_2-{\upmu}_1\right)}^2 $$

where N is the sample size required per group, SD is the pooled standard deviation, α is the SD, K is the constant (7.9 denotes 80% power and 5% significance), and (μ_2_ – μ_1_) is the difference in vegetable intake (grammes) between groups.

### Inclusion and exclusion criteria

Infants will be eligible for this study if they are born term (37 weeks or greater), of normal growth/weight, and have no known food allergies, chronic diseases, or medical conditions. Infants must not have started complementary feeding and must live in Auckland. Mother’s proficiency in English is needed given the requirement to complete assessment tools as part of the secondary outcomes. Participants will be excluded if they demonstrate repeated non-adherence to key study procedures or are not able to complete the 4-week intervention period.

### Setting

The study, data collection and analyses will take place in Auckland, New Zealand, from May 2019 to October 2021. Auckland is the major commercial city of New Zealand, with an ethnically diverse population of 1.6 million and around 21 thousand child births recorded in 2018 [51].

### Infant foods

Freeze-dried infant foods are specifically developed for the study by the researchers. According to study specifications, these are manufactured by FreshAs° who specialise in the production of high-quality freeze-dried vegetables and fruits and are based in Auckland, New Zealand. Vegetables and fruits are selected based on availability, infant nutrition guidelines, total sugar content per 100 g, and colour. A Dietitian and Speech and Language Therapist who specialises in child feeding and development will conduct recipe testing in the laboratory. This confirms that to reconstitute the powders into age-appropriate infant purées, each sachet requires the addition of 50 ml of water (except potato and green bean which require 80 ml). The food is then heated in the microwave for three 20-s intervals, stirred in between, and then cooled for 10 min until it is at a safe temperature for infants to consume. Final instructions are provided on recipe cards to participants.

The foods provided to the intervention group will be less sweet than controls (< 4 g total sugar per 100 g) and made from vegetables only (Table 1). The control group receives foods that will be sweeter than intervention (> 4 g total sugar per 100 g) and fruit based, resembling commercial infant foods that are currently available on the market. Following nutrition guidelines, the study will provide a range of different coloured vegetables, including green, purple, white, and orange. However, it will not be possible to provide an orange-coloured food for the intervention group due to limitations to the freeze-drying method and high sweetness profile of available orange-coloured vegetables. Therefore, the intervention group will be offered a second green food, named “Kākāriki”, which is the Māori translation for green and so should resonate with New Zealand families.

**Table 1:** Infant foods allocated to the intervention and control group

Food name	Food ingredient (%)^a^	
	Intervention group	Control group
Green purée	Spinach (80%), potato (20%)	Apple (90%), spinach (10%)
White purée	Potato (100%)	Pear (100%)
Purple purée	Beetroot (70%), potato (30%)	Pear (98%), beetroot (2%)
Orange purée	Not applicable	Pumpkin (100%)
Kākāriki purée	Green bean (100%)	Not applicable

^a^Total dry weight is 8 g per sachet; wet weight is 80 g.

At baseline, mothers will receive enough food to provide up to three meals per day for the duration of the intervention and will be able to access additional sachets from the Massey University Human Nutrition Research Unit (HNRU) as required. To ensure infants meet iron intake requirements while participating in the study, mothers with an infant starting complementary feeding at 6 months of age will be especially encouraged to provide their own meat (beef, fish, or chicken) during the intervention following recipe cards and videos provided.

### Feeding protocol

**Fig. 2:**
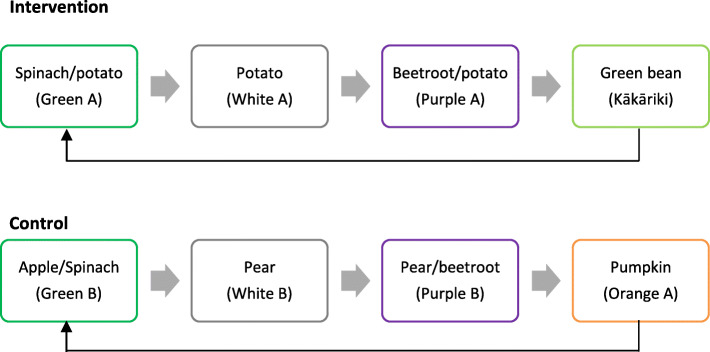
Food rotations for intervention and control group, either per day (recording days) or per meal (other days). Foods will be labelled by colour and none list the ingredients but mothers are reassured that the foods align with New Zealand infant feeding guidelines. The code A or B act as identifiers for researchers

Mothers will determine the timing of meals according to their own schedule. However, they will feed the foods according to a prescribed recipe and colour rotation to improve standardisation and ensure infants are exposed to a variety of flavours (Fig. 2). On recording days (the first and last 4 days) mothers must only provide one colour per day, starting with green. On other days, colours rotate per meal. No meat is provided during the first 4 days to standardise infants’ very first tastes. To cater to individual infant needs, mothers can provide as many meals as they need per day.

The rotation order of foods offered for the intervention group is determined by sugar content, starting with the first food containing the lowest sugar per 100 g. This should help to expose infants to more bitter flavours first. The order is matched to the intervention group to improve standardisation and ensure vegetables, such as spinach, will be introduced at the same time where possible. Instructions on feeding style and environment will be provided, for example, “let baby lead”, “use a spoon”, and “choose a quiet time without distractions”. At 9 months of age, each group will be offered a brassica (broccoli), green leafy vegetable (spinach) then sweet fruit (pear) over 3 consecutive days (one per day). These will be labelled meal A, meal B, and meal C, respectively. Infants should not have had solid food within 1 h prior to trying the meal. Assessment of intake of each food should determine whether a “vegetables first” approach to complementary feeding results in higher intake of a brassica or green leafy vegetable at 9 months of age compared to a control group.

### Education session

To standardise the feeding sessions as much as possible, all mothers will receive a 40-min education session on appropriate infant feeding practices during their first visit, delivered by the primary investigator who is also a New Zealand registered Dietitian. Topics include identifying the signs of developmental readiness to start complementary feeding, responsive feeding, and appropriate breast/formula milk feeding. Instructions for the study protocol and food diary will also be discussed in detail. A series of educational resources, including three infant feeding fridge magnets designed for the study will be provided during the session and mothers will be encouraged to visit the study website to view additional resources. After completion of the 4-week intervention, all mothers receive the Ministry of Health *Eating for Healthy Babies and Toddlers/Ngā kai tōtika mō te hunga kōhungahunga* [52] and Beef and Lamb New Zealand *Fuelled by Iron* [53] pamphlets to assist infants’ transition to family foods.

### Randomisation, blinding, and concealed allocation

Eligible and consented mother-infant pairs will be randomly allocated to either the intervention or control group, having been stratified by infant gender and using a random number generator by the research trials manager who is not involved in the study design and outcome analysis. In accordance to block randomisation with a 1:1 allocation, for every block of eight participants, four will be allocated to each arm of the trial, with the block size concealed until the primary endpoint is analysed. Allocation concealment will be ensured, as the release of the randomisation code occurs only until the participant has been recruited into the trial, which is after consent and baseline measurements have been completed. After assignment, researchers will not be blinded given their necessary involvement in the food product development and subsequent familiarity with food characteristics (e.g. colour). While participants will be provided with an information sheet that lists all the types of fruit and vegetables included on the study, they will be blinded to the specific foods allocated to them as foods are labelled generically (e.g. “Green A” rather than “spinach/potato”). They will also be unaware of the RCT design, so do not know specific differences between feeding regimes. In an emergency where breaking of the study blind is needed, plans are in place for the principal investigator to reveal the treatment assignment for a given participant.

### Data collection

**Fig. 3:**
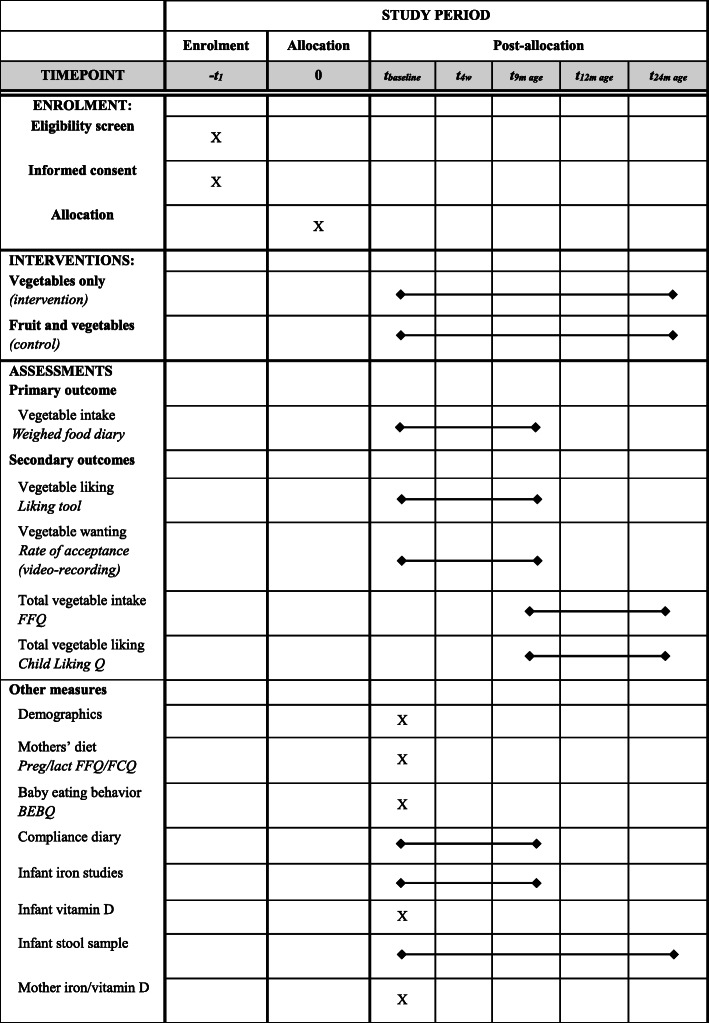
Schedule of enrolment, interventions, and assessments. According to the SPIRIT statement: Defining Standard Protocol Items for Clinical Trials. *t,* timeline; *w,* week; *FFQ,* food frequency questionnaire; *preg,* pregnancy; *lact*, lactation; *FCQ,* food choices questionnaire; *Q,* questionnaire

Mothers will be emailed the links to the online demographics, baby behaviour, and pregnancy/lactation food frequency questionnaires to complete at home before their first visit to the HNRU at which other baseline data (infant anthropometry and blood samples) will be collected. The 4-week intervention and primary data collection (mother reported infant vegetable liking and intake, video-recorded meals) will take place in the infant’s home environment from May 2019 to October 2021. Follow-up data (anthropometry and/or blood samples) will be collected at 9, 12, and 24 months of age at the HNRU; home visits will be available as required. In the instance where visits are not possible, mothers will be asked to provide current infant growth measurements (following standardised instructions given by the researcher) and food diaries by email or post. Virtual meetings using Zoom or Skype will be available for additional support. Additional child vegetable/fruit liking and food frequency questionnaires will be completed by mothers at home when their infant is 9, 12, and 24 months old. To the extent possible, we will continue to collect data for all outcome measures for participants who discontinue or deviate from the intervention protocols, while anticipating and planning for missing data. Given the potential for this cohort to provide valuable data for future early life nutrition research, additional outcome measures will be collected. These include mothers’ iron status/vitamin D status (at baseline), infants’ vitamin D status (at baseline), and infant stool samples for microbiota analysis (pre- and post-4-week intervention, and all follow-ups). Such data may be useful to assess maternal iron status 6 months post-partum or infant vitamin D status and links to the microbiome. Table 2 outlines the primary study outcome measures and testing methods. Figure 3 provides the schedule of enrolment, intervention, and assessment.

**Table 2:** Summary of the study outcome measures and methods

Variables	Method
Primary outcomes	
Vegetable liking	Liking tool (Appendix A). Completed by mother at home
	Rate of acceptance video coding tool. Mother video-records meals. Independent researcher codes infant behaviours
	Child vegetable and fruit liking questionnaires. Completed online by mother
Vegetable intake	Weighed food diary. Completed by mothers at home
	FFQ. Completed online by mother
Blood analysis^a^	
Infant iron studies^b^	“Heel prick” test by phlebotomist at Massey University or home
Infant vitamin D	“Heel prick” test by phlebotomist at Massey University or home using *Whatman card*
Mother iron/vitamin D	Venous blood sample collection by phlebotomist at Massey University
Other measures	
Infant anthropometry	Head circumference: paper measuring tape, widest part of head, three measures, select largest measurement to the nearest 0.1 cm; length: infant length board; weight: infant electronic weighing scales, no nappy; measured by researcher at Massey University or participant home
Demographics	Completed online by mother
Mother diet	Pregnancy and lactation FFQ and FCQ. Completed online by mother
Baby eating behaviour	Baby eating behaviour questionnaire [54]. Completed online by mother
Compliance	2-min check-in questionnaires. Completed online by mother

^a^Analysed by LabTests, Auckland

^b^Serum ferritin, C-reactive protein (CRP), haemoglobin

*FFQ* food frequency questionnaire, *FFQ* food frequency questionnaire, *FCQ* food choices questionnaire

### Weighed food diary

Mothers will complete a 4-day weighed food diary during the first and last 4 days of the 4-week intervention to primarily measure infant food intake (grammes). In alignment with other studies [33–39, 55–57], this will involve weighing the food before and after consumption using provided digital scales and completing the food record sheet. Estimated spills or food fallen on the bib will be recorded. The greater amount of food eaten may indicate a greater preference or acceptance for that food. In addition, the diary collects data for breast/formula milk feeding, meat consumption, and medication/supplements. Mothers can report factors that may have affected infant feeding each day (e.g. teething, unwell).

The diary is designed specifically for this study and includes a liking tool to assess the degree that infants like the study foods (see Liking tool). Combining these tools into one booklet should reduce participant burden and improve data collection. Detailed and visually appealing instructions on the feeding environment and protocol, such as food rotation and video-recorded meals, are included. Food diaries for each group differ only by the allocated foods mentioned; for example, instructions for the orange food do not feature for the intervention group diary. Week 1 and week 4 diaries also differ slightly by the removal of irrelevant instructions; for example, the week 4 diary excludes the instruction “After the first four days…”. A similar 3-day weighed food diary will be provided at 9 months of age but includes the option to list any other vegetables or fruits eaten that day.

Weighed food records are considered the best estimate food intake for children aged 0.5 to 4 years [58]. The use of 4- and 3-day weighed food diaries should help to reduce participant burden that is typically associated with those of greater durations (e.g. a 7-day food diary) without compromising accuracy [59, 60]. These durations also fit well with the protocol; for example, three tests meals offered over three consecutive days at 9 months of age is appropriately measured by a 3-day food diary.

### Liking tool

The liking tool for our study is adapted from the new in-home validated elaborate method to assess infant liking of vegetables [41]. The tool asks mothers to use a form to report positive and negative behaviours for each spoon offered for the first nine spoons in their home environment, then rate how much they thought their infant liked the food after each triplet of spoons (i.e. 3 x 3 spoons), then again at the end of the meal. After piloting the tool with a small group of New Zealand mothers and with further expert consultation it was agreed that a pictorial 5-point liking scale (1 = dislikes very much to 5 = likes very much), and a list 10 positive and 10 negative behaviours per spoon, which mothers can tick as they occur would reduce participant burden. The greater percentage of positive behaviours recorded may indicate greater liking, while an average score of at least 4 on the liking scale should indicate that the food is liked. It is required that a researcher provides mothers with additional guidance on feeding environment, feeding style to adopt, when to terminate the meal (three consecutive refusals), and the list of cues related to liking/disliking. These are adapted to align with New Zealand infant feeding guidelines [1], for example, the instruction to minimise verbal communication during feeding is removed as this conflicts with recommendations to interact with and talk to the child at meal times.

### Video coding tool

**Fig. 4:**
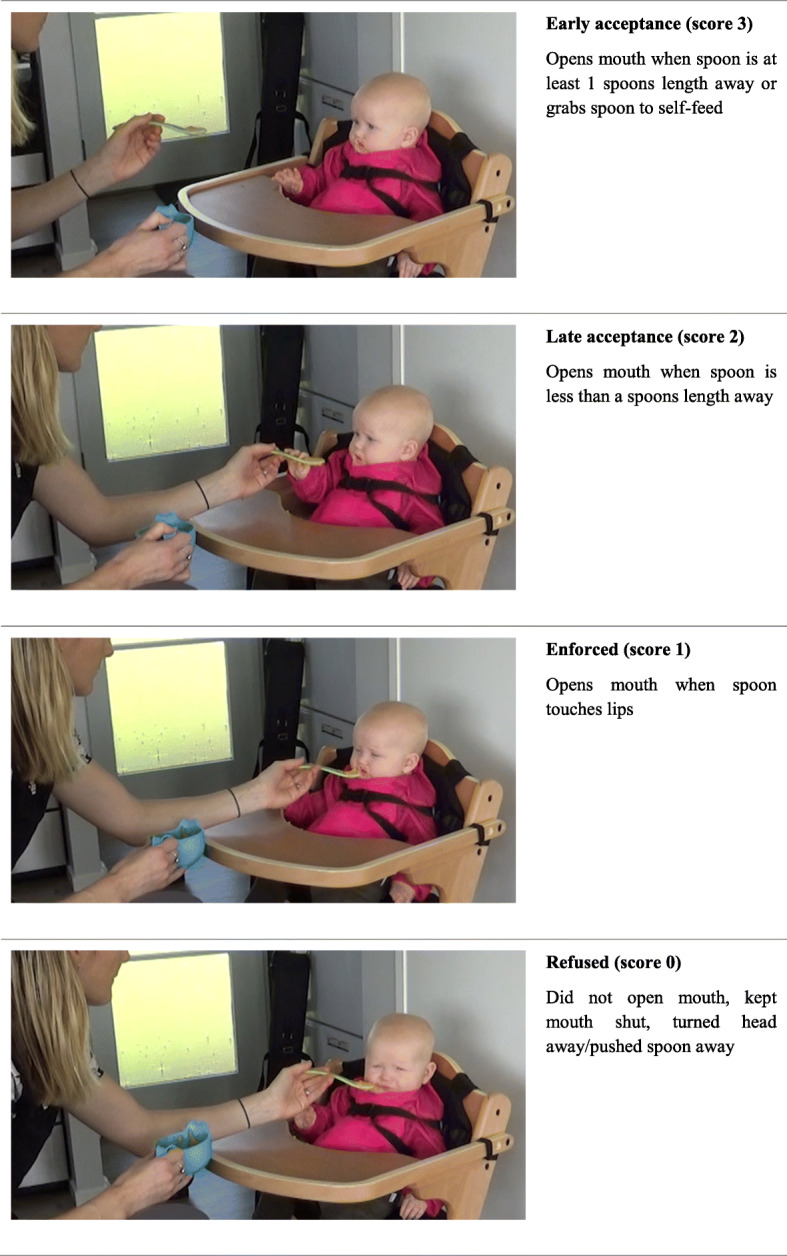
Video coding for rate of acceptance. Images provided with written permission from the participating subjects

During the intervention, mothers will be asked to video record their infant trying each study food at home for the first time in week 1, then again in week 4 without the researcher present. This provides a total of eight videos. When infants turn 9 months old, mothers will video record their infant at home trying meal A, B, and C. Each participant will be given access to their own secure online shared OneDrive file to upload their videos. To assess rate of acceptance, the videos will be coded using the coding tool described in Fig. 4. Rate of acceptance is the judgement made on how readily the infant accepts the spoon offered and is a measure of wanting. This outcome measure will be included because liking (hedonic drive) and wanting (motivation to eat) are considered interconnected components of food reward and pleasure but can be measured separately [61–63]. The coding tool is based on prior studies [40, 64], but with further consultation with the researchers, the addition of “grabs spoon to self-feed” as an indicator of early acceptance, as well measurable spoon distances are included to improve accuracy and standardisation. Coding will be completed by an independent researcher to reduce bias.

### Questionnaires

A total of nine online questionnaires specifically developed for this study are to be completed by mothers via the survey software tool Qualtrics™. Researchers will check all answers for completeness.

#### Eligibility questionnaire

A 5-min eligibility questionnaire ensures that only infants that meet the inclusion criteria will be included.

#### Demographics questionnaire

A 10-min demographics questionnaire collects information at baseline on mother and infant date of birth, ethnicity, medical history, geographical location, mothers’ education level, and parity.

#### Pregnancy/lactation food frequency questionnaire (FFQ) and food choices questionnaire (FCQ)

As the mother’s diet may predict infant liking and intake of vegetables, a FFQ asking about mothers’ intake of vegetables and fruit during pregnancy and lactation is adapted from an existing New Zealand validated FFQ [65]. An accompanying FCQ gains further insight on other dietary habits, such as foods avoided. These questionnaires are peer-reviewed by three experts in the nutrition and dietetics field and pilot tested with 14 new mothers. Their feedback informed minor wording changes to increase readability and acceptability, thus improving content validity; each questionnaire requires 15–20 min to complete at baseline.

#### Food frequency questionnaire (FFQ) [66]

An abbreviated online version of a validated complementary feeding food frequency questionnaire for mothers to complete in about 15 min when infants are 9, 12, and 24 months old collects data on infants’ vegetable intake and variety. The questionnaire is abbreviated because only items relating to vegetables, fruit, and meat and alternatives are included to meet study objectives and reduce participant burden. Additional items relating to milk feeding history are added for the purpose of this study. The tool asks mothers to enter the total number of times and typical amount of a specified food (e.g. “Beans and peas”) eaten by the infant in the last 4 days using free text. Check boxes are used for milk feeding items. The data will be interpreted so that the greater variety and frequency of a vegetable observed, the greater intake of that vegetable. As the tool is validated to assess nutrient intake in infants, this data may be useful for further analysis of nutrient intakes when following a “vegetables first” approach to complementary feeding.

#### Fruit and vegetable liking questionnaire

An online child vegetable and fruit liking questionnaire for mothers to complete when infants are 9, 12, and 24 months old collects data on vegetables and fruits tried and liked. It features visually appealing images and the same liking scale used in the adapted infant liking tool. Scores of 4 or above indicate a greater liking for the food. Content validity is confirmed by four experts in the nutrition and dietetic field and five mothers not participating in the study. To improve completion, the questionnaire is divided into two online questionnaires, with one focusing on vegetables and the other on fruit; the estimated completion time is 10 min each.

#### Baby Eating Behaviour Questionnaire (BEBQ)

As infant feeding behaviour characteristics could impact results, an online version of the validated BEBQ is to be completed [54]. This is an 18-item questionnaire with 17 items measuring four aspects of infant feeding behaviour and one item measuring general appetite before starting complementary feeding. Response options range from never = 1 to always = 5. Scoring involves calculating mean scores for each subscale with higher mean scores indicating greater presence of the feeding behaviour. The four themes are enjoyment of food (e.g. My baby seems contented while feeding), food responsiveness (e.g. My baby frequently wants more milk than I provide), slowness in eating (e.g. My baby takes more than 30 min to finish feeding), and satiety responsiveness (e.g. My baby get full up easily). This questionnaire should take 10 min to complete. It is expected that infants who enjoy food less, are less food responsive, eat slower, are less sensitive to internal cues of satiety, and have a smaller overall appetite may be more difficult to feed and perhaps less willing to accept vegetables specifically, compared to infants with the opposite traits.

### Compliance to study protocol

A 2-min check-in compliance questionnaire collects data on how many days the intervention food was offered, other food or drink consumed other than provided in the study, medications, and factors that may affect infant feeding (e.g. teething). This will be emailed to participants on the completion of each intervention week. Mothers will be contacted by email or telephone at 9-, 12- and 24-month follow-ups and an e-card will be sent on child birthdays to improve sample retention. Mothers will receive a personalised baby bag, kitchen scales, visually appealing infant feeding resources, and petrol vouchers at no cost to them in order to improve participation rates. The video footage from mothers’ first video recordings will be checked for compliance and quality, with corrections (e.g. lighting, camera angle, environment) administered by the researcher as required.

### Website

A study website is designed for participants to use when wishing to register, complete the eligibility questionnaire, learn more about the study, contact the researchers, and access exclusive infant resources. The use of a New Zealand website domain, the Massey University logo, and a visually appealing study banner may improve credibility and recruitment. The website acts as a platform for distributing participant education and resources, including seven videos, three downloadable fridge magnets, a food safety flyer, and three meat purée recipe cards with accompanying videos. These are based on Ministry of Health infant feeding guidelines and created using design software (Canva, Photoshop, and Adobe Premier Pro). Images and video content are obtained from a photography/video shoot with written consent from the participating subjects, purchased from Shutterstock® or taken from Creative Commons. Using online surveys, each infant feeding resource is peer-reviewed by six experts in infant nutrition and 20 mothers. Feedback informed revisions; for example, it was recommended to use brighter colours for the recipe cards to improve engagement.

### Adverse events

In the rare case that an allergic reaction to the infant foods, participants will be instructed to stop feeding the food and may withdraw from the trial and provided a referral to a doctor. Reports of gastrointestinal symptoms or other side effects will be further investigated by the trial registered Dietitian and the infant’s health will be monitored closely for the duration of the intervention. If the adverse event persists, withdrawal from the trial and/or a referral to a doctor for further investigation is expected. All adverse events will be recorded, and mothers offered telephone/email support. If the participant needs to make a claim to Accident Compensation Corporation (ACC) due to an adverse event and the claim is not accepted, then the researcher will initiate processes to ensure they receive appropriate compensation.

### Blood sampling, stool collection, and analysis

As part of screening for and monitoring infant health and iron status, capillary blood samples will be taken from infants by a registered phlebotomist using a standard “heel prick” test at baseline, after the intervention, and at the 9-month follow-up. The focus on monitoring iron status reflects concerns for meeting iron requirements during the complementary feeding period [24], with iron deficiency having consequences that are more serious and irreversible than in adults [1, 67]. Thus, serum will be used for the analysis of serum ferritin and C-reactive protein (CRP) at LabTests (Auckland). Haemoglobin will be measured using HemoCue in our HNRU laboratory. If Haemoglobin is <110 g/L, the participant will be notified and given a referral letter to see their doctor.

Additional blood and stool collection will be taken in the interest of future research. At baseline, venous blood samples will be collected from mothers and will be stored for later analysis of iron and vitamin D. Whatman cards will collect infant serum for a future study investigating infant vitamin D at baseline. For microbiome research purposes, mothers will collect a stool sample from their infant before and after the 4-week intervention, then at 9, 12, and 24 months of age.

### Dissemination of results

During each visit, mothers will receive their infant’s growth measurements and blood haemoglobin result. The remaining blood results will be provided on request. Once available, participants who completed the intervention will receive a summary of findings in lay language, and whether they were assigned to the intervention or control. Results will be presented at national and international scientific conferences, prepared for publication in peer-reviewed journals, and circulated to the media and should be of interest to a range of audiences including families, health professionals, district health boards, academics, and primary health organisations. Individuals with substantive contributions to the design, conduct, interpretation, and reporting of the study will be recognised by granting authorship on the final relevant report.

### Data handling

Name and address details will be kept in a separate Microsoft Excel spreadsheet, and will include progress check boxes in order to track and schedule follow-ups. All entries will be cross-checked by another member of the research team. The video content to be stored on the shared storage platform OneDrive will be protected by Citrus Consulting Group Limited, a high security data management and storage company in New Zealand. Only the researcher and the participant have access to the designated video folder labelled by subject number. All data will be stored safely under confidential conditions and archived for at least 5 years, and only the researchers will have access and permissions to the final intervention dataset.

### Statistical analysis

Statistical analysis will be performed using IBM SPSS version 25.0 (IBM Corp. Released 2017, IBM SPSS Statistics for Windows Version 25.0. Armonk, NY, IBM Corp.). Data will be cleaned and checked for coding errors and completeness. To assess if data is normally distributed, the Kolmogorov-Smirnov and Shapiro-Wilk tests and normality plots will be used. Data that is not normally distributed will be transformed using log transformations to improve normality. Mean (standard deviation) and median (25, 27 percentiles) will be used to report normal and non-normal data, respectively. Transformed data will be reported at geometric mean (95% CI) following back transformation, and categorical data as frequencies.

Baseline characteristics of infants and mothers will be compared across intervention groups using the independent *t*-test or Mann-Whitney test for continuous variables (e.g. infants’ age, weight, breastfeeding duration, and mothers’ age), and chi-square tests for categorical variables (e.g. gender, milk feeding type, and mothers’ education).

For the primary analysis at 9 months, Independent t-test or Mann-Whitney test will be used to assess differences in vegetable intake and other food acceptance variables, depending on data distribution.

Additional analyses will be performed to investigate if the intake of intervention foods and mothers-rated liking and rate of acceptance after 4 weeks are correlated (using Pearson’s or Spearman’s correlations depending on data distribution).

Given the nature of the intervention, per protocol (PP) analyses will be conducted. Although this may compromise the integrity of randomisation and reduce sample size, PP should help address dilution effects of non-compliance/missing data that is associated with intention-to-treat analysis. PP may also provide more information on the efficacy of the treatment. Reasons for excluding patients (e.g. loss to follow-up, major deviations from the protocol) will be fully reported. All statistical tests will be two-tailed with an alpha value of P < 0.05, and corrections applied for multiple comparisons where indicated.

## Discussion

Despite the known health benefits of a diet rich in vegetables and fruit [2, 68, 69], children’s vegetable consumption remains lower than recommended worldwide [8–12]. Introducing vegetables at the beginning of complementary feeding may be a promising strategy to promote vegetable intake in children, both immediately and later in life [25]. However, the few studies that have investigated this approach vary considerably in methodology and are limited by small sample sizes, short duration, and/or poor ecological validity [33–35, 37–39]. The main purpose of this randomised controlled trial is to address this research gap by determining whether exposure to vegetables only during the first 4 weeks of complementary feeding increases later intake and liking of vegetables in infants, compared to a control group which includes both fruit and vegetables.

Strengths of this study include the randomisation of infants, longitudinal design that follows infants until 24 months of age, use of diverse and complementary methods to assess outcome measures, a relatively large sample size, improved ecological validity (e.g. conducted in the home environment for the outcomes of interest), and using a control that mimics current practice. Possible limitations include difficulties for families to adhere to study procedures and remain engaged for up to 24 months of age. Since this is a convenience sample, participants are self-selecting and likely interested in nutrition and health with the potential to generate bias in the participant recruitment. However, extensive measures have been put in place to mitigate these, such as weekly compliance questionnaires, offering home visits/virtual meetings, providing incentives (free infant foods, petrol vouchers), and blinding mothers to the foods. In addition, time has been spent to develop a feeding protocol that is easy to follow (e.g. use of engaging colours, visual aids, and clear instructions) and facilitated by evidenced-based infant feeding support.

We anticipate that this trial will provide important insights into the cause-effect relationship between a “vegetables first” approach to complementary feeding and vegetable liking and intake in children. To our knowledge, this is the first study to investigate such causality of association in the New Zealand context. If successful, this study may help inform future updates of national and international infant feeding recommendations and lead to practical advice for caregivers and health professionals wanting to improve vegetable consumption in children.

## Supplementary Information

**Additional file 1.** Information Sheet.

**Additional file 2.** Consent Form.

**Additional file 3.** Liking Tool.

**Additional file 4.** SPIRIT 2013 Checklist: Recommended items to address in a clinical trial protocol and related documents*.

## Data Availability

Data and tools will be made available on request and in accordance with the ethics approval for the study.

## Abbreviations

BEBQ: Baby Eating Behaviour Questionnaire
FFQ: Food Frequency Questionnaire
FCQ: Feeding Choices Questionnaire
HNRU: Human Nutrition Research Unit
