# Supplementary material for: Impact of a “vegetables first” approach to complementary feeding on later intake and liking of vegetables in infants: a study protocol for a randomised controlled trial
Source: Trials. 2021 Jul 26;22:488. doi: 10.1186/s13063-021-05374-7 (PMC8314593; doi:10.1186/s13063-021-05374-7)
Supplement: Supplementary file 1 — Additional file 1. Information Sheet. [file 13063_2021_5374_MOESM1_ESM.docx]

**Vegetables as first foods for babies study**

## INFORMATION SHEET

You are invited to take part in the *Vegetables as first foods for babies study* which is looking at the impact of a vegetables first approach to complementary feeding on infant food preferences.

The researchers are as follows:

| 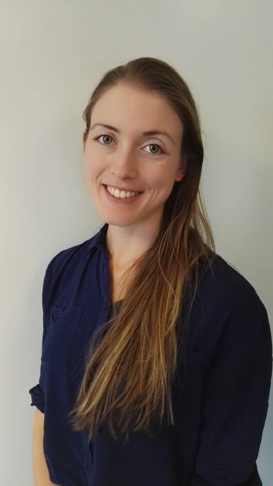 | 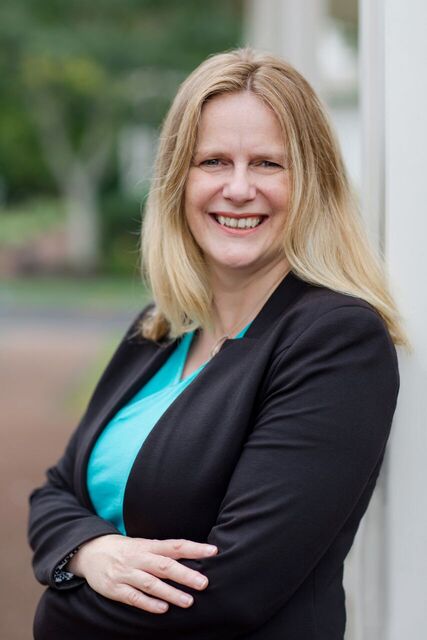 | 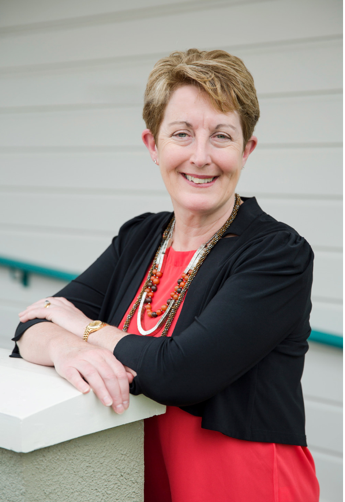 |
| --- | --- | --- |
| **Lead researcher** | **Supervisor** | **Supervisor** |
| **Jeanette Rapson**  PhD candidate, registered dietitian  School of Sport, Exercise and Nutrition  0210773419 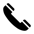 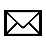vegesfirst@massey.ac.nz | **Dr Cath Conlon**  Senior Lecturer  School of Sport, Exercise and Nutrition  09 414 0800 ext. 43658 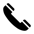 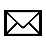[C.Conlon@massey.ac.nz](mailto:C.Conlon@massey.ac.nz) | **Dr Pam von Hurst**  Associate Professor  School of Sport, Exercise and Nutrition  09 414 0800 ext. 43657 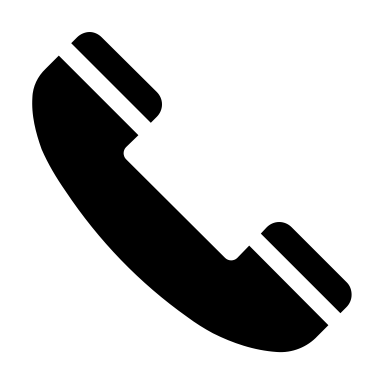 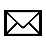[P.R.vonHurst@massey.ac.nz](mailto:P.R.vonHurst@massey.ac.nz) |

Whether or not you take part is your choice. If you do not want to take part, you do not have to give a reason. If you do want to take part now, but change your mind later, you can pull out of the study at any time.

This Participant Information Sheet will help you decide if you would like to take part. It sets out why we are doing the study, what your participation would involve, what the benefits and risks to you might be, and what would happen after the study ends. We will go through this information with you and answer any questions you may have. You do not have to decide today whether or not you will participate in this study. Before you decide you may want to talk about the study with other people, such as family, whānau, friends, or healthcare providers. Feel free to do this.

If you agree to take part in this study, you will be asked to sign the Consent Form. You will be given a copy of both the Participant Information Sheet and the Consent Form to keep.

This document is seven pages long. Please make sure you have read and understood all the pages.

If you have any further question, concerns or complaints about the study at any stage, please do not hesitate to contact our lead researcher:

**Jeanette Rapson***Lead researcher*

Tel: 0210773419

email: [vegesfirst@massey.ac.nz](mailto:vegesfirst@massey.ac.nz)

Website: www.vegesfirststudy.com

**What is the study about?**

In New Zealand, complementary feeding is recommended to start at around 6 months. Suggested first foods include vegetables, fruits, meat, and commercial infant foods including baby rice.

Introducing vegetables early in life may help children like and eat more vegetables later. It is important that children eat plenty of different vegetables and fruits to provide the vitamins, minerals and dietary fibre needed for their growth and development.

Few studies have investigated different combinations of vegetables and fruits during complementary feeding, especially in New Zealand. The aim of this study is to compare two different feeding regimes which differ in the types of vegetables and fruits they provide. We will also examine the mother’s diet during pregnancy and lactation as this may have some impact on the taste preference of their child.

Our results may better inform current infant feeding recommendations in New Zealand and worldwide.

**Who can take part?**

We are looking for 120 mothers and their baby to volunteer to participate in this study.

To take part in all parts of this study, your baby should be:

- 4-6 months of age
- healthy and born at term gestation (37+ weeks)
- with no known allergies
- with no chronic diseases or medical conditions that may affect abilities to participate in the study
- at a stage where they have not started eating solids
- living in the Auckland region

**What will my participation involve?**

Your participation involves visiting our Human and Nutrition Research Unit, giving a blood sample, having measurements taken for your baby (growth, heel prick blood sample and faecal) completing dietary and lifestyle questionnaires, and video-recording your infant trying foods for the first time.

If you decide to take part in this study after you have read and had time to consider this information sheet, you will be asked to complete a screening questionnaire to ensure that you meet the study’s eligibility criteria. If eligible, you will be invited to take part in the study and asked to sign a consent form. You will then be sent a series of dietary and lifestyle questionnaires to complete at home.

Total time involved for this study is **10 hours over a period of 24 months.** This time is divided as follows:

Each visit takes about 1 hour (total of 4 hours). Questionnaires each take about 10-30 minutes to complete (total of 2.5 hours). It is not anticipated that infant feeding will take any longer than what is usual for you, however, additional time will be needed to complete infant feeding diaries and video recordings (5-10 minutes per day).

**When your baby is around 4 months old**

A researcher will make an appointment with you and your baby to visit the Human Nutrition Research Unit at Massey University in Albany at a time that suits. We will provide reserved free parking, appropriate bathroom facilities and private spaces for feeding. This visit will take about **1 hour.**

During this visit, we will ask you to:

- Meet with a New Zealand registered dietitian to discuss any questions you may have about the study and infant feeding.
- Tell us more information about your diet and lifestyle.
- Have a small blood sample taken from yourself by a qualified phlebotomist (about 20ml which is equivalent to 4 teaspoons) to measure your iron and vitamin D levels.
- Have a small blood sample taken from your baby by qualified phlebotomist using a ‘heel prick’ test to measure their iron and vitamin D levels (please see [what does a heel prick involve?](#heel))
- Have your baby’s length, weight and head circumference measured.
- Provide a small faecal sample from your baby to assess their faecal microbiota composition (please see [what does a faecal sample involve?](#stool))
- Collect your infant feeding pack, which includes the foods, equipment and instructions needed during the 4-week trial.
- Discuss with a researcher about video recording your baby’s feeding sessions using a device of your choice, e.g. mobile phone, video camera.

You and your baby will be randomly allocated one of two infant feeding regimes that differ by the types of vegetables and fruits they provide. You will not be told which feeding regime you have been assigned to; however, the feeding regimes include vegetables and fruits that are recommended for babies starting solids. All babies will receive approved age-appropriate foods that enable them to meet recommended dietary guidelines (please see ‘what will the foods be like?’).

**During the 4-week trial**

When your baby is ready to start solids (around 4-6 months of age), we will ask you to:

- Notify the researcher that you have started/ready to start complementary feeding.
- Feed the allocated foods to your baby for a period of 4-weeks alongside breastmilk or infant formula. You will also be asked to please not provide any other foods or beverages except water and meat (please see [what will the foods be like?](#food))
- Video record the first time you feed the allocated foods to your baby, and then at least two additional feeding sessions during the 6-weeks.
- Complete a daily weighed food diary (please see [what does a weighed food diary involve?)](#diary)
- Each week you will be asked to compete an online 2-minute check-in questionnaire to see how you are going.

**After the 4-week trial**

You will no longer need to feed the allocated foods and can resume your normal family diet. A researcher will contact you to arrange a time to meet you and your baby at the research unit at Massey University Albany (home visits available on request). This visit will take about **30 minutes.**

During this visit, we will ask you to:

- Have a small blood sample taken from your baby by qualified phlebotomist using a ‘heel prick’ test to measure their iron levels.
- Have your baby’s length, weight and head circumference measured.
- Provide a small faecal sample from your baby to assess their faecal microbiota composition.
- Provide your baby’s infant feeding diary, videos and return any borrowed equipment.

**When your baby is 9 months old**

When your baby is 9 months of age, a researcher will contact you to arrange a time to meet you and your baby at the research unit at Massey University Albany (home visits available on request). This visit will take about **30 minutes.**

During this visit, we will ask you to:

- Have a small blood sample taken from your baby by qualified phlebotomist using a ‘heel prick’ test to measure their iron levels.
- Have your baby’s length, weight and head circumference measured.
- Provide a small faecal sample from your baby to assess their faecal microbiota composition.
- Video record your baby trying a vegetable food that we will provide.
- Complete dietary questionnaires for your baby.

**When your baby is 12 & 24 months old**

When your baby is 12 & 24 months of age, a researcher will send you online questionnaires to complete then arrange a time to meet you and your baby at the research unit at Massey University Albany (home visits available on request). This visit will take about **30 minutes.**

During the visit, we will ask you to:

- Have your baby’s length, weight and head circumference measured.
- Provide a small faecal sample from your baby to assess their faecal microbiota composition.

**FAQ**

**What does the ‘heel prick’ test involve?**

Iron is extremely important for infant brain development and growth. Iron deficiency can also cause taste disturbances, which may affect your baby’s liking and intake of foods. It is vital that your baby has good iron status for the study outcomes and their health. Babies also need good levels of vitamin D for growth and development.

The common way to measure and assess iron and vitamin D status in babies is to do a ‘heel prick’ test. This involves making a pinprick puncture in one heel of your baby to collect a small blood sample. This is completed by a qualified phlebotomist and is then sent to a laboratory to be analysed.

You can ease any distress for your baby by cuddling and feeding them and making sure they are warm and comfortable. Your baby’s foot will also be warmed by a sock prior to the heel prick test to minimise discomfort. There is a small risk of infection at the puncture site, however strict hygiene and safety procedures will be followed to minimise this risk.

**What does a faecal sample involve?**

The human microbiota consists of a wide variety of microorganisms such as bacteria, viruses and fungi. The common way to measure the composition of an infant’s microbiota is to collect a small faecal (stool) sample and then analyse it in a laboratory. We will ask you to use a collection kit that we provide with easy to follow instructions.

**What will the foods be like?**

You will receive freeze-dried vegetables and fruit powders that are conveniently made into a smooth puree by adding water. These have been commercially prepared, packaged, and developed especially for this study by a food manufacturer. There are no preservatives, gluten, dairy or nuts added to these foods. They are easy to store and travel with. All foods meet recommended guidelines and safe for baby.

The types of vegetables include: spinach, beetroot, green bean, broccoli, pumpkin and potato. The types of fruits include: apple and pear.

Foods do not contain meat. However, meat is encouraged as an iron-rich food during the study. Support will be given on how to prepare and provide meat foods.

**What does a weighed food diary involve?**

It is important for us to understand how much of the foods your child eats and their liking of it. An easy way to do this is to weigh (in grams) the amount of leftover food using food kitchen scales, then record this in the diary. We will provide a set to use during the trial. Alternatively, you can freeze the leftover foods and we will collect this from you to complete the measurement.

In this diary, you will need to rate your baby’s liking of the given food using a simple rating scale (1=likes very much to 9=dislikes very much), then describe anything that might have affected you baby’s feeding that day. You will be given training and support on how to complete this.

What are the possible benefits and risks of this study?

Direct benefits of participating in this study include:

- Free infant nutrition and feeding support from a registered dietitian throughout the study period.
- Free nutritious infant foods during the 4-week trial.
- You will receive feedback on you and your baby’s individual blood results, and your baby’s growth measurements. You will be advised if any of the blood results are outside of a normal range and advised to seek advice from your general medical practitioner (GP). We will provide you with a copy of your results to give to your GP.
- An increased awareness and knowledge of the processes involved in research by actively participating in it, and a satisfaction in knowing that you are contributing to nutrition knowledge in the community.
- At the end of the study you will receive a DVD with memorable video clips of your child that were made during the research.
- You will receive a brief report summarising the main findings of the project via mail or email.

Foreseeable risks, adverse-effects and discomforts that you may encounter by taking part in this study are minimal but could include possible infection from the site in which blood is drawn and there may be some minor bruising at this site as well. Your baby may also feel some discomfort from the heel prick test. These discomforts will be managed by the presence of a qualified phlebotomist who will be available to assist you should you require it. While all foods provided by the study will be safe, age-appropriate and approved first foods for babies, it is possible that your baby may not like or tolerate the food. A record of all adverse events will be monitored and maintained throughout the course of the study.

Unless otherwise arrange, all follow up visits will be conducted at your home with consent and if deemed safe for the researcher and participant. We will ask if you have any cultural issues or concerns around taking these measurements before they are taken. For example, if you are Maori and consider the head as tapu (sacred), we will ask for your permission before touching the infant’s head.

**Does it cost money to participate?**

There is no monetary cost to you, the participant, for taking part in this study. You will be reimbursed for travelling costs with a $20 voucher following your first visit to the Massey University Human and Nutrition Research Unit (Albany). If you choose to visit our research unit for the follow-up visits, you will be given a $20 voucher following each visit.

**What are my rights?**

You are under no obligation to accept this invitation. If you decide to participate, you have the right to:

- decline to answer any particular question;
- withdraw from the study (specify timeframe);
- ask any questions about the study at any time during participation;
- provide information on the understanding that your name will not be used unless you give permission to the researcher;
- be given access to a summary of the project findings when it is concluded;
- ask for the recorder or video to be turned off at any time during the interviews or video recordings.

**What if something goes wrong?**

If physical injury results from your participation in this study, you should visit a treatment provider to make a claim to ACC as soon as possible. ACC cover and entitlements are not automatic and your claim will be assessed by ACC in accordance with the Accident Compensation Act 2001. If your claim is accepted, ACC must inform you of your entitlements, and must help you access those entitlements. Entitlements may include, but not be limited to, treatment costs, travel costs for rehabilitation, loss of earnings, and/or lump sum for permanent impairment. Compensation for mental trauma may also be included, but only if this is incurred as a result of physical injury.

If your ACC claim is not accepted you should immediately contact the researcher. The researcher will initiate processes to ensure you receive compensation equivalent to that to which you would have been entitled had ACC accepted your claim.

**Data management**

The data will be used only for the purposes of this project and no individual will be identified. Only the investigators and administrators of the study will have access to personal information and this will be kept secure and strictly confidential. Participants will be identified only by a study identification number.

Results of this project may be published or presented at conferences or seminars. No individual will be able to be identified.

At the end of this study the list of participants and their study identification number will be disposed of. Any raw data on which the results of the project depend will be retained in secure storage for 16 years, after which it will be destroyed.

Samples will be stored separately and only Dr Cath Conlon, Owen Mugridge and Jeanette Rapson will have access to these records. You will be given the option for samples donated by yourself or your child to be stored for use in future research studies.

**What happens if I change my mind?**

You are able to stop participating in the study at any time and will be compensated accordingly for your time. Further you are welcome to discuss any concerns you have with the research team at any time, and you have free access to your data. If you withdraw from the study all of the data that was related to you will be destroyed.

The foods and support from a dietitian will not be available to any participant after the study. The outcomes of this study may inform future recommendations enable further investigation in a long-term intervention study.

The study data will be stored at a secure location at Massey University Albany Campus. Electronic data and records will be the responsibility of the Principal investigator. All data will be kept for 10 years, at which point it will be destroyed using University Security methods for removal of confidential material. At the completion of the study all biological samples collected will be disposed of using established methods for discarding biological waste. Any participant can request to have their remaining blood sample returned to them.

You may hold beliefs about a sacred and shared value of all or any tissue samples removed. The cultural issues associated with storing your tissue should be discussed with your family/whanau as appropriate. There are a range of views held by Maori around these issues; some iwi disagrees with storage of samples citing whakapapa and advise their people to consult prior to participation in research where this occurs. However, it is acknowledged that individuals have the right to choose.

We anticipate that the results of this study will be published in a peer-reviewed journal within 12 months of completing the study. Participants are welcome to discuss the findings of this study with the researchers at any time.

**Who do I contact for more information or if I have concerns?**

If you have any questions, concerns or complaints about the study at any stage, you can contact researchers in the study.

You can contact the following researchers:

Jeanette Rapson, Dr Cath Conlon or Associate Professor Pam von Hurst. Contact details are at the beginning of this information sheet.

##### Committee Approval Statement

*This project has been reviewed* and approved by the Massey University Human Ethics Committee: Southern A, *Application SOA 18/56. If you have any concerns about the conduct of this research, please contact Dr Lesley Batten, Chair, Massey University Human Ethics Committee: Southern A, telephone + 64 6 356 9099 x 85094, email humanethicsoutha@massey.ac.nz .*
