# Supplementary material for: Impact of a “vegetables first” approach to complementary feeding on later intake and liking of vegetables in infants: a study protocol for a randomised controlled trial
Source: Trials. 2021 Jul 26;22:488. doi: 10.1186/s13063-021-05374-7 (PMC8314593; doi:10.1186/s13063-021-05374-7)
Supplement: Supplementary file 2 — Additional file 2. Consent Form. [file 13063_2021_5374_MOESM2_ESM.pdf]

Default Question Block

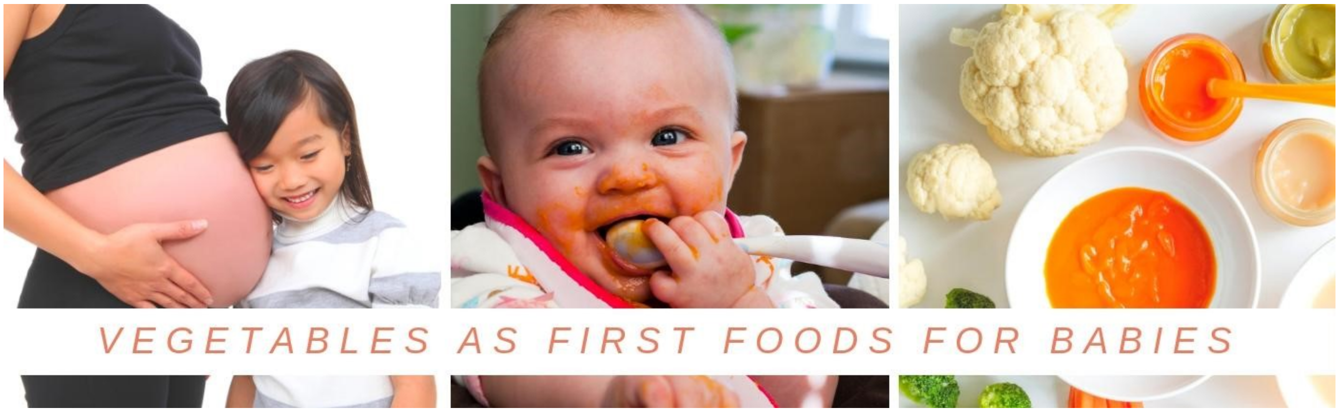

Participant Consent Form

I have read the Information Sheet and have had the details of the study explained to me.

My questions have been answered to my satisfaction, and I understand that I may ask further questions at any time.

I agree for my baby and I to participate in the study under the conditions set out in the Information Sheet.

○ Agree

Parent/Caregiver

First name

Last name

Baby

First name

Last name

Date of consent:

	Day	Month	Year
Please Select:			

Are you willing to be contacted regarding future research projects within the School of Sport Exercise and Nutrition? Your name and email address will be saved in a secure location. You will be sent periodic newsletters regarding research studies within the School. You can opt out of this newsletter at any time.

- Yes
- No

Powered by Qualtrics
