# Supplementary material for: Impact of a “vegetables first” approach to complementary feeding on later intake and liking of vegetables in infants: a study protocol for a randomised controlled trial
Source: Trials. 2021 Jul 26;22:488. doi: 10.1186/s13063-021-05374-7 (PMC8314593; doi:10.1186/s13063-021-05374-7)
Supplement: Supplementary file 3 — Additional file 3. Liking Tool. [file 13063_2021_5374_MOESM3_ESM.docx]

Behaviour form: first 3 spoons

If they **refuse** the spoon 3 times in a row, please stop feeding.

Please complete this **WHILE feeding** **Meal A.**

For **each spoon,** which behaviours did your baby show? *Tick all that apply*

|  | **When the spoon is approaching** | | **When the food is**  **in the mouth** | | **Refused** |
| --- | --- | --- | --- | --- | --- |
| **Spoon number** | **Positive** | **Negative** | **Positive** | **Negative** |  |
| **1** | - Opened mouth - Leaned forward - Reached out for food - Looked interested - Took spoon to feed self | - Closed mouth - Turned head away - Looked away - Arched back/pulled body away - Pushed spoon away | - Smiled - Happy sound, e.g. giggles, Mmm, oohs - Excited, e.g. waves arms, kicks legs - Swallowed food - Licked lips | - Frowned/nose wrinkled - Shivered/stiffened - Spat food out - Cried/fussed - Pushed spoon away |  |
| **2** | - Opened mouth - Leaned forward - Reached out for food - Looked interested - Took spoon to feed self | - Closed mouth - Turned head away - Looked away - Arched back/pulled body away - Pushed spoon away | - Smiled - Happy sound, e.g. giggles, Mmm, oohs - Excited, e.g. waves arms, kicks legs - Swallowed food - Licked lips | - Frowned/nose wrinkled - Shivered/stiffened - Spat food out - Cried/fussed - Pushed spoon away |  |
| **3** | - Opened mouth - Leaned forward - Reached out for food - Looked interested - Took spoon to feed self | - Closed mouth - Turned head away - Looked away - Arched back/pulled body away - Pushed spoon away | - Smiled - Happy sound, e.g. giggles, Mmm, oohs - Excited, e.g. waves arms, kicks legs - Swallowed food - Licked lips | - Frowned/nose wrinkled - Shivered/stiffened - Spat food out - Cried/fussed - Pushed spoon away |  |

My infant likes it a lot

Please **rate** how much baby likes **Meal A**? *Please tick one*

| 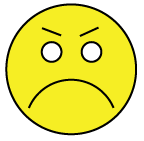  Dislikes very much | 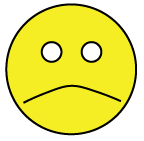  Dislikes | 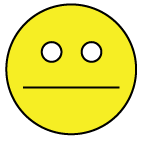Neither like nor dislike | 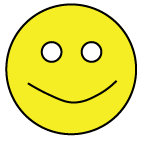  Likes | 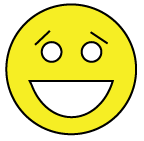  Likes very much |
| --- | --- | --- | --- | --- |
|  |  |  |  |  |

…next 3 spoons

If they **refuse** the spoon 3 times in a row, please stop feeding.

For **each spoon,** which behaviours did your baby show? *Tick all that apply*

|  | **When the spoon is approaching** | | **When the food is**  **in the mouth** | | **Refused** |
| --- | --- | --- | --- | --- | --- |
| **Spoon number** | **Positive** | **Negative** | **Positive** | **Negative** |  |
| **4** | - Opened mouth - Leaned forward - Reached out for food - Looked interested - Took spoon to feed self | - Closed mouth - Turned head away - Looked away - Arched back/pulled body away - Pushed spoon away | - Smiled - Happy sound, e.g. giggles, Mmm, oohs - Excited, e.g. waves arms, kicks legs - Swallowed food - Licked lips | - Frowned/nose wrinkled - Shivered/stiffened - Spat food out - Cried/fussed - Pushed spoon away |  |
| **5** | - Opened mouth - Leaned forward - Reached out for food - Looked interested - Took spoon to feed self | - Closed mouth - Turned head away - Looked away - Arched back/pulled body away - Pushed spoon away | - Smiled - Happy sound, e.g. giggles, Mmm, oohs - Excited, e.g. waves arms, kicks legs - Swallowed food - Licked lips | - Frowned/nose wrinkled - Shivered/stiffened - Spat food out - Cried/fussed - Pushed spoon away |  |
| **6** | - Opened mouth - Leaned forward - Reached out for food - Looked interested - Took spoon to feed self | - Closed mouth - Turned head away - Looked away - Arched back/pulled body away - Pushed spoon away | - Smiled - Happy sound, e.g. giggles, Mmm, oohs - Excited, e.g. waves arms, kicks legs - Swallowed food - Licked lips | - Frowned/nose wrinkled - Shivered/stiffened - Spat food out - Cried/fussed - Pushed spoon away |  |

My infant likes it a lot

Please **rate** how much baby likes **Meal A**? *Please tick one*

| 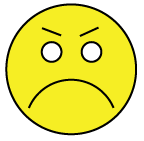  Dislikes very much | 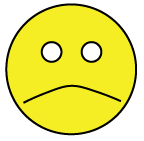  Dislikes | 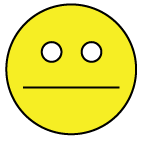Neither like nor dislike | 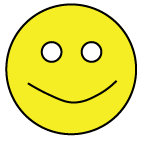  Likes | 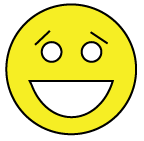  Likes very much |
| --- | --- | --- | --- | --- |
|  |  |  |  |  |

…next 3 spoons

If they **refuse** the spoon 3 times in a row, please stop feeding.

For **each spoon,** which behaviours did your baby show? *Tick all that apply*

|  | **When the spoon is approaching** | | **When the food is**  **in the mouth** | | **Refused** |
| --- | --- | --- | --- | --- | --- |
| **Spoon number** | **Positive** | **Negative** | **Positive** | **Negative** |  |
| **7** | - Opened mouth - Leaned forward - Reached out for food - Looked interested - Took spoon to feed self | - Closed mouth - Turned head away - Looked away - Arched back/pulled body away - Pushed spoon away | - Smiled - Happy sound, e.g. giggles, Mmm, oohs - Excited, e.g. waves arms, kicks legs - Swallowed food - Licked lips | - Frowned/nose wrinkled - Shivered/stiffened - Spat food out - Cried/fussed - Pushed spoon away |  |
| **8** | - Opened mouth - Leaned forward - Reached out for food - Looked interested - Took spoon to feed self | - Closed mouth - Turned head away - Looked away - Arched back/pulled body away - Pushed spoon away | - Smiled - Happy sound, e.g. giggles, Mmm, oohs - Excited, e.g. waves arms, kicks legs - Swallowed food - Licked lips | - Frowned/nose wrinkled - Shivered/stiffened - Spat food out - Cried/fussed - Pushed spoon away |  |
| **9** | - Opened mouth - Leaned forward - Reached out for food - Looked interested - Took spoon to feed self | - Closed mouth - Turned head away - Looked away - Arched back/pulled body away - Pushed spoon away | - Smiled - Happy sound, e.g. giggles, Mmm, oohs - Excited, e.g. waves arms, kicks legs - Swallowed food - Licked lips | - Frowned/nose wrinkled - Shivered/stiffened - Spat food out - Cried/fussed - Pushed spoon away |  |

My infant likes it a lot

Please **rate** how much baby likes **Meal A**? *Please tick one*

| 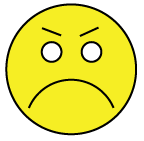  Dislikes very much | 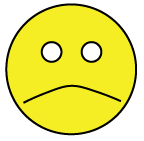  Dislikes | 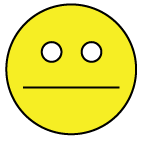Neither like nor dislike | 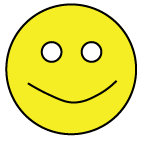  Likes | 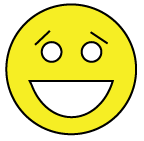  Likes very much |
| --- | --- | --- | --- | --- |
|  |  |  |  |  |

How much did your baby like Meal A?

Please rate **at the END of the meal.**

| 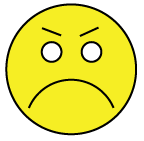  Dislikes very much | 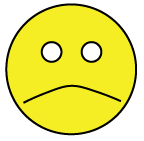  Dislikes | 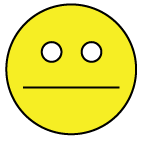Neither like nor dislike | 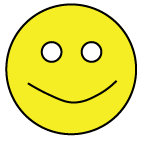  Likes | 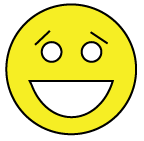  Likes very much |
| --- | --- | --- | --- | --- |
|  |  |  |  |  |
